# Breast lump in a patient with Type 1 Diabetes

**DOI:** 10.1080/17571472.2016.1163937

**Published:** 2016-03-22

**Authors:** Aditi Sharma, Sarah Ali, Senan Devendra

**Affiliations:** ^a^ Specialist Training Registrar in Endocrinology, London Deanery (North West Thames), London, UK; ^b^ Acute Medicine & Endocrinology, Watford General Hospital, West Hertfordshire Hospitals NHS Trust, Watford, UK

## Why this matters to me?

The detection of a breast lump causes understandable fear of breast cancer in both the patient and the clinician. Patients usually first present to their general practitioner with the finding. Although majority of palpable breast lumps are benign, a thorough history, examination and timely onward specialist referral to the breast team for further investigations is essential to confirm the diagnosis, and therefore alleviate patient concerns. Here, we take the opportunity to report a case of diabetic mastopathy – an important yet-not-so-widely known complication of long-standing Type 1 diabetes that presents as a palpable breast lump in a young patient.

## Key messages

(1) Diabetic mastopathy should be one of the considered differentials of a breast lump in patients with long-standing Type 1 Diabetes.
(2) There are identifiable risk factors in the patient’s history associated with diabetic mastopathy.
(3) Diabetic mastopathy is not linked with breast cancer.
(4) No surgical treatment is required. Unnecessary excision due to misdiagnosis can lead to extensive recurrence at sites of excision putting patients at risk of undue psychological stress.
(5) It should be managed with conservative surveillance with proper counselling and periodic imaging follow-up.

## Case history

A 37-year-old multiparous Caucasian lady with a 22-year-history of Type 1 Diabetes Mellitus presents with a 1 month history of a painless lump in her right breast. She has no family history of breast cancer and is a non-smoker. Her recent HbA1c was 11.4% DCCT; 101 mmol/mol (IFCC). She has diabetic nephropathy and has had previous laser therapy for proliferative diabetic retinopathy. On examination, there was a mobile, non-tender firm nodule in the right breast medial to the nipple, with no associated peau d’orange nor dimpling. There was no discharge from the nipple nor was there any cervical or axillary lymphadenopathy. She was referred by the GP as a two-week wait pathway to the local breast clinic for an urgent assessment of the lump.

### Ways to think of the problem

#### What could account for the breast signs?

There are many potential causes of breast lumps which are broadly classified as benign or malignant. Majority of breast lumps are benign and the commonest causes include: fibrocystic disease, fibroadenoma, breast abscess and mastitis. Diabetic mastopathy is a condition, first described in 1984, characterised by benign proliferation of fibrous tissue of the breast.[1] It is also known as ‘sclerosing lymphocytic lobulitis’ or simply ‘lymphocytic mastitis’ due to its characteristic histology consisting of marked fibrosis and lymphocytic infiltrates.[1, 2] It is most commonly encountered in pre-menopausal women with long-standing Type 1 Diabetes mellitus (T1DM), usually after a decade of initial diagnosis, although there are a small number of case reports of its occurrence in Type 2 diabetics and non-diabetic patients with other autoimmune conditions.[3–6] The condition has also been rarely reported in men.[7, 8]

Diabetic mastopathy constitutes <1% of benign breast lesions. Its reported prevalence ranges from 0.6 to 13% of women with T1DM, with prevalence likely to be underestimated because most young diabetic women do not routinely have breast examinations.[9]

The exact pathogenesis of diabetic mastopathy is still unclear and likely to be multifactorial. As the lymphoid infiltrates on histology are predominantly B-cell, in contrast to T-cell predominance seen in other types of non-diabetic mastitis, this supports an underlying autoimmune aetiology. This is further illustrated by its association with autoimmune thyroid disease, such as Hashimoto’s thyroiditis, and similarities with lymphocytic mastopathy of Sjogren’s syndrome and systemic lupus erythematous.[6, 10, 11]

#### What investigations are necessary to make a diagnosis of Diabetic mastopathy?

As illustrated in this case study, the patient usually presents to their general practitioner with a breast lump which is clinically indistinguishable from breast cancer, that is, a non-tender, ill-defined, hard mass on palpation. These can commonly be single or multiple, unilateral or bilateral at presentation.[2]

When a female patient first presents with a breast lump, the immediate concern is to exclude a diagnosis of breast cancer. To this effect, all referrals with a lump, to breast clinic, from primary care (in England) should be seen within two weeks, whereby further investigations are warranted to exclude breast cancer (Table 1).

**Table 1.** Diabetic mastopathy: summary of investigations, their typical findings and limitations.[2, 12–14]

Investigation	Result	Limitations
Ultrasound	Irregular hypoechoic, microlobulated mass(es) with extensive acoustic shadowing	Indistinguishable to breast cancer
Mammogram	Dense glandular tissue. No microcalcifications, no spicules nor architectural distortions seen	Indistinguishable to breast cancer. Findings can be equivocal and sometimes misleading
MRI breast	T1- and T2-weighted and STIR images may show an irregular mass which has heterogeneous low signal intensity	Heterogeneous spotty enhancement may also be a feature of breast carcinoma
	3-D FLASH images: mass shows poor enhancement in the early phase with a gradual increase in the degree of enhancement. In the delayed phase, the mass shows ‘heterogeneous spotty enhancement’	Variable non-diagnostic findings
Fine needle aspiration	Firm resistance experienced during the back and forth motion of the needle is stronger than that in other conditions	Little diagnostic value – depends on whether diagnostic tissue is obtained
Core needle excision biopsy	Histology is diagnostic of diabetic mastopathy	Reoccurrence of lesions at sites of excision biopsy are reported
	Histology shows: Breast tissue with (i) densely hyalinised stroma in which there are occasional prominent enlarged stromal cells. (ii) dense keloidal fibrosis (iii) dense aggregates of lymphocytes around ducts (lymphocyctic ductitis), around lobules (lymphocytic lobulitis) and around small blood vessels (lymphocytic perivasculitis) (iv) epitheloid fibroblast embedded in the dense collagenous matrix	
	*Epitheloid fibroblasts and lymphocytic perivasculitis are the most characteristic of diabetic mastopathy	

Ultrasound and mammography findings are often non-specific. On ultrasound, one typically reports a discrete mass with extensive acoustic shadowing whilst mammograms show homogenous dense glandular tissue without any architectural distortions. Images of both these modalities are unhelpful and could simulate breast cancer.[2, 12]

The diagnostic value of fine needle aspiration depends on whether diagnostic material has been obtained. Diabetic mastopathy contains little cellular material resulting in multiple difficult-to-pass needle attempts.[12]

Due to the indistinguishable findings of these initial investigations, most patients will require a core needle biopsy, which is the diagnostic investigation of choice. The characteristic histopathology hinges the diagnosis of diabetic mastopathy, as originally defined by Seidman et al. [13]

There has been an increasing interest in using MRI modality to investigate diabetic mastopathy. However, described MRI findings have been variable.[14] More studies are required to validate the use of MRI as a diagnostic tool for this condition.

#### Are there any key factors that may be associated with diabetic mastopathy?

*In most case reports, patients have co-existing microvascular complications of diabetes. Kudva et al. indeed showed, as evident in our case study, that there was a significantly higher prevalence of diabetic neuropathy and diabetic retinopathy in those with diabetic mastopathy* [15] (*Table* 2)*. They failed to show any link with the degree of glycaemic control.*

**Table 2.** Risk factors associated with diabetic mastopathy.

• Type 1 Diabetes • Pre-menopausal state • Pre-existing autoimmune disorders • Pre-existing microvascular complications • Longer duration of Type 1 Diabetes (>10 years)

Our present case was typical to that reported in other literature, that is, a premenopausal woman with poorly controlled T1DM, and the pre-existence of microvascular complications.

#### Is diabetic mastopathy linked with breast cancer?

This is the area that gives both the clinicians and the patients their greatest concerns. Diabetic mastopathy is a benign condition that is not considered pre-malignant. Despite there being a few case reports of concominant breast cancer diagnosed in patients who also have diabetic mastopathy, there is no conclusive evidence to suggest a link between the two conditions, and that the two conditions occurring together may be coincidental rather than causal, in view of the higher prevalence of breast cancer compared to diabetic mastopathy. This stresses the need for appropriate initial differentiating investigations preventing future clinical dilemmas.

The benign nature of diabetic mastopathy is shown by Kudva et al. who followed up patients, identified in their study with histologically confirmed diabetic mastopathy, between a median of 6.2 and 9.9 years. None of the patients developed breast carcinoma or lymphoma during this period.[15]

Additionally, till date there have been no confirmed reported cases of malignant transformation of diabetic mastopathy.

#### How should our patient be managed?

##### 1. Short term management

Once a patient has presented with a breast lump, clinically there is nothing concrete to differentiate between diabetic mastopathy and breast cancer. For this reason, specialist referral to the breast clinic for further investigations are highly warranted and recommended. The current limitations in imaging means that core needle biopsy is inevitable. Once diabetic mastopathy has been confirmed histologically, no treatment is required at this stage, other than reassurance.

##### 2. Long term management

Although diabetic mastopathy is benign, routine follow-up is recommended annually as recurrence can occur at sites of excision.[16] Currently, there is no suggestion that diabetic mastopathy is associated with an increased incidence of breast carcinoma, however, further longer follow-up studies are required.

### Outcome of the case

Our patient underwent an ultrasound and mammography of the right breast which raised suspicion of malignancy. FNA cytology was inadequate, hence a core needle biopsy was performed which confirmed the benign features suggestive of diabetic mastopathy. Patient was reassured and is under annual review by the breast team.

## Governance

This work is based on a case discussed and overseen by an Ealing multidisciplinary group of the Integrated Care Programme.

## Disclosure statement

All authors declare that the answer to the questions on your competing interest form are all [No] and therefore have nothing to declare.

## Copyright

The Corresponding Author has the right to grant on behalf of all authors and does grant on behalf of all authors, an exclusive licence (or non-exclusive for government employees) on a worldwide basis to the International Medical Publishing Group, and its Licensees to permit this article (if accepted) to be published in LJPC editions and any other IMPG products and to exploit all subsidiary rights, as set out in our copyright and exclusive licence.

## References

[CIT0001] Soler NG, Khardori R (1984). Fibrous disease of the breast, thyroiditis and cheiroarthropathy in type 1 diabetes mellitus. Lancet.

[CIT0002] Ghosh S, Collier A, Milne A (2008). Diabetic mastopathy. Pract. Diabet. Int..

[CIT0003] Tsung JS, Wang TY, Lin CK (2005). Diabetic mastopathy in type 2 diabetes mellitus. J. Formos. Med. Assoc..

[CIT0004] Sotome K, Ohnishi T, Miyoshi R (2006). An uncommon case of diabetic mastopathy in type 2 diabetes mellitus. Breast Cancer.

[CIT0005] Kojima T, Kammori M, Hashimoto M (2003). Diabetic mastopathy in an advanced elderly woman with insulin-dependent type 2 diabetes mellitus. Breast Cancer.

[CIT0006] Valdez R, Thorson J, Finn WG (2003). Lymphocytic mastitis and diabetic mastopathy: a molecular, immunophenotypic, and clinicopathologic evaluation of 11 cases. Mod. Pathol..

[CIT0007] Cavazza A, Nigrisoli E, Tinterri C (1997). Male diabetic mastopathy: description of a case. Pathologica.

[CIT0008] Ely KA, Tse G, Simpson JF (2000). Diabetic mastopathy. A clinipathologic review. Am. J. Clin. Pathol..

[CIT0009] Shaffrey JK, Askin FB, Gatewood OM (2000). Diabetic fibrous mastopathy: case reports and radiologic–pathologic correlation. Breast J..

[CIT0010] Ríos G, Peredo RA (2010). Lymphocytic mastitis preceding Sjögren’s syndrome. P. R. Health Sci. J.

[CIT0011] Schwartz IS, Strauchen JA (1990). Lymphocytic mastopathy: an autoimmune disease of the breast. Am. J. Clin. Pathol.

[CIT0012] Mak CW, Chou CK, Chen SY (2003). Case report: diabetic mastopathy. Brit. J. Radiol..

[CIT0013] Seidman JD, Schnaper LA, Phillips LE (1994). Mastopathy in insulin requiring diabetes mellitus. Hum. Pathol..

[CIT0014] Sakuhara Y, Shinozaki T, Hozumi Y (2002). MR imaging of diabetic mastopathy. Am. J. Roentgenol..

[CIT0015] Kudva YC, Reynolds C, O’Brien T (2002). “Diabetic mastopathy”, or sclerosing lymphocytic lobulitis, is strongly associated with Type 1 Diabetes. Diabet. Care.

[CIT0016] Camuto PM, Zetrenne E, Ponn T (2000). Diabetic mastopathy: a report of 5 cases and a review of literature. Arch. Surg..

